# The Treatment of Exposed Pulps in Filling Teeth

**Published:** 1875-09

**Authors:** Samuel Welchens

**Affiliations:** Lancaster, Pa.


### ARTICLE IV.

### *The Treatment of Exposed Pulps in Filling Teeth.*

BY SAMUEL WELCHENS, D. D. S., LANCASTER, PA.

Read before the District Dental Society of the Seventh Judicial District of the State of N. Y., at Rochester, N. Y., June 1st and 2nd 1875.

In the treatment of the subject in hand it is not our purpose to go into a detailed and scientific investigation of the nature and character of that portion of the dental organ known to science as the pulp, but more commonly denominated "the nerve." Its composition, character, and general bearing upon the nutrition, texture and vitality of the tooth should be so thoroughly understood by every dental practitioner as that such an investigation here would be entirely superfluous. If we go back to the earlier stages of dentition, and trace the most interesting of all formative processes through its changes and developments to the end, and then contemplate the causes and ravages of disease upon the dense tissue until the pulp is involved in the disorder, we should have such a vivid appreciation of its nature and character as to render it necessary that its treatment when exposed should partake of the greatest care, and be fraught with the highest scientific attainments.

The pulp is not the only source of vitality to the tooth, nor does its death necessarily involve the complete death of the organ. But its relation to the general structure and growth of the tooth is of such a character as to render its life an essential factor and principle to such an extent as to make its preservation a leading feature in the treatment of the tooth, which, of course, calls for the highest scientific ability upon the part of the operator.

It is my purpose to treat the subject in hand under three heads, namely:

1. The liability of striking the tubuli when excavating a cavity preparatory to filling, and the treatment.

2. The exposure of the pulp proper, either by caries or the instrument, and its treatment.

3. The extirpation of the pulp when too much diseased for conservative treatment, and the filling of the cavity.

THE DENTAL TUBULI AND FIBRILLÆ.

It is well known to the careful student of dental tissue that the calcification of the teeth is not the work of a day. The process is a slow and tedious one, and so delicate in its function as to render it subject to the slightest aberrations of the nutritive process, thus causing imperfections of structure, weakness of tissue, and often serious malformation. These are sometimes seen upon the surface, but often are hidden in the body or substance of the tooth when it seems to be perfectly developed. The process of vitrification commences with the dentine, hardening inward from the inner surface of the enamel until the pulp has been reduced to its normal size or limit. After this there is no change in the structure of the tooth, except that which is caused by caries or a slight hardening of the tissue in after life, whereby the pulp is somewhat diminished and the fibrillæ perceptibly shortened by the ossification of the tubuli. Hence we find that in young teeth the fibrillæ which enter the tubuli, and are elongations of the substance of the pulp, and especially in teeth that, through some constitutional or nervous weakness, become frail and chalky, spread outward boldy, approaching the surface of the dentine and sometimes even penetrating the enamel, so that they are easily reached either by caries or an instrument when the disorder is slight and the cavity exceedingly superficial.

We are apt to say of such teeth when treating them tha "they are all nerve." It is to this class of teeth and this kind of structure that attention is here called.

The elongation of the fibrillæ above referred to is the result of one of two causes; either a malformation of the dentine in the process of calcification whereby minute fissures are left in its structure, which are usually filled with the albuminous material of the pulp, or through some disturbance of nervous energy during the process of dentition, rendering the whole structure of the teeth frail and chalky, thus causing the extension of the tubuli a necessary provision in the nutritive process by producing a stimulant which is designed by nature to supply with animal matter what they failed to receive in enduring texture.

We find a similar provision of nature in the vegetable kingdom. A plant or tree, when in active vegetation, throws out fibrils from the main branches of the root, thus obtaining an immense absorbing surface just when the most nourishment is needed. After the season of active vegetation has passed and the plant has borne its fruit, the fibrils die and decay off, and the body of the root receives the elaborated sap, the material for which they contributed so much in gathering up the crude elements from the earth.

The fibrillæ of the teeth seem to hold the same function, though in structure and character they are vastly different. In the case of children, when, in consequence of rapid growth, the demand for nutrition in all the tissues is greater than in the adult, and especially when the teeth are weak in texture, the elongation of the fibrillæ affording a larger nutritive surface seems to be necessary. And then when time and age have done their work of perfect development, and the teeth are rendered more dense, the pulp containing all the elements of dentine is diminished, the fibrillæ very frequently through the process of calcification are hardened into dentine and disappear altogether.

When a tooth containing these extended tubuli is attacked by caries, the disease need not extend far below the surface in order to strike the fibrillæ and involve the pulp. In excavating the cavities in teeth of this character, the tubuli are often opened with the instrument, and the fibrillæ laid bare, and even portions of them cut off, when there is no more pain than in ordinary sensitive dentine. There may be no soreness immediately resulting than occasionally slight trouble from thermal changes, or the inconvenience of an unpleasant consciousness that the tooth is there, but a discolored tooth, in a year or two after the operation has been performed, will tell the tale. How often are we not confronted with devitalized teeth in the mouths of some of our most confiding patients, when upon removing the filling we find the pulp dead from an operation which we regarded at the time it was performed as perfectly secure and successful, and the failure of which we could trace to no other cause than filling upon the exposed fibrillæ. The danger of thus encountering those vital tissues should be kept steadily in mind by the operator, and when a cavity in the kind of teeth above described extends to any depth below the surface, care should be taken that it receive proper treatment before the filling is inserted.

For the purpose of closing up the mouths of the tubuli, and thus protecting the exposed fibrillæ, Canada Balsam is often used. I am not prepared to commend or condemn this treatment. My objection to it is that it is a vegetable substance containing no medicament of much value, and as a simple stoppage of the mouth of the tubuli it may be improved upon by a substance less liable to stagnation and disintegration.

I think the most scientific and the most reliable treatment that can be instituted is to apply upon a pledget of cotton either diluted carbolic acid or creosote as an antiseptic, and then cover the bottom of the cavity with a thin coating of oxychloride of zinc or Guillois' cement, and fill upon it as soon as it is hard. We have in this treatment not only an antiseptic and a good firm covering for the mouths of the tubuli, but the cement being an astringent and absorbent both the effusions and gases which may be the result of the injury, slight though they be, will be quickly taken up, and thus a perfect and enduring substitute is at once afforded.

In my practice I have no failures with the above treatment.

TO BE CONTINUED.

